# Enhancing ascitic fungal infection diagnosis through next-generation sequencing: a pilot study in surgical ICU patients

**DOI:** 10.3389/fcimb.2024.1441805

**Published:** 2024-11-01

**Authors:** Sara Posadas-Cantera, Negin Mehrbarzin, Simon Wetzel, Hanna Goelz, Lampros Kousoulas, Stefan Utzolino, Georg Häcker, Mohamed Tarek Badr

**Affiliations:** ^1^ Institute of Medical Microbiology and Hygiene, Faculty of Medicine, Medical Center - University of Freiburg, Freiburg, Germany; ^2^ Centre for Inherited Metabolic Diseases (CMMS), Karolinska University Hospital, Solna, Sweden; ^3^ Institute of Clinical Chemistry and Laboratory Medicine, Faculty of Medicine, Medical Center - University of Freiburg, Freiburg, Germany; ^4^ Department of General and Visceral Surgery, Faculty of Medicine, Medical Center - University of Freiburg, Freiburg, Germany; ^5^ BIOSS Centre for Biological Signaling Studies, University of Freiburg, Freiburg, Germany; ^6^ IMM-PACT-Program, Faculty of Medicine, University of Freiburg, Freiburg, Germany

**Keywords:** surgery, ascitic fluid infections, mycobiome, intensive care unit, clinical microbiology, next-generation sequencing, molecular diagnostics, fungal infections

## Abstract

**Objectives:**

Ascites, often associated with critical pathologies such as liver cirrhosis or bowel perforation, can be complicated by fungal infection, increasing mortality especially in intensive care settings and demanding rapid diagnosis and adequate treatment. Traditional microbiological diagnostic methods have limited sensitivity in accurately identifying fungal pathogens in ascitic fluid. Alternative diagnostic methods may offer important insights to enable guiding of antifungal therapy and refining empirical treatment strategies. The objective of this study was to evaluate the potential of next-generation sequencing methods to identify specific fungal pathogens responsible for ascitic fluid infections.

**Methods:**

We prospectively collected 50 ascitic fluid samples from ICU patients with suspected ascites infection. In addition to standard culture-based microbiological testing, an ascitic fluid aliquot underwent fungal DNA isolation and was analyzed by next-generation sequencing (NGS) methods for identification of fungal species.

**Results:**

Of 50 ascitic samples collected, five samples showed growth of *Candida* spp. in culture. After DNA isolation and ITS2 PCR, detectable amplification was achieved in 10 samples. Sequencing of the 50 patients’ samples identified facultative pathogenic fungi in 19 patients. In 15 cases, culture alone would not have permitted the identification of all facultative pathogenic fungi. The identification of fungal DNA by sequencing was significantly associated with poor patient outcome and a number of clinical parameters.

**Conclusions:**

Our results show a higher sensitivity for NGS-based diagnostic methods in the identification of ascitic fluid fungal infections compared to culture-based diagnostics. This may be beneficial especially for patients in a critical care setting, who have an increased prevalence of comorbidities and high mortality. The implementation of such methods in standard diagnosis will require increased standardization of the workflows and interpretation of the sequencing results with respect to patients’ clinical picture.

## 1 Introduction

Ascites is the abnormal accumulation of fluid within the abdominal cavity (Runyon, 1994), frequently associated with cirrhotic liver disease and other conditions such as heart failure, tuberculosis, pancreatitis, and cancer. In intensive care unit (ICU) settings, ascitic fluid infection emerges as a severe complication (Vincent et al., 2009; Arvaniti et al., 2010), correlating with increased morbidity and mortality rates and representing one of the primary infections in such critical environments (Montravers et al., 1996; Marshall and Innes, 2003).

While bacterial infections of the ascitic fluid are regularly considered and identified, spontaneous fungal peritonitis (SFP) and ascitic fungal infections are an alarming yet often overlooked complication, usually in hospitalized patients (Brown et al., 2012; Hwang et al., 2014). The infection is typically diagnosed by having an elevated neutrophil count (>250 cells/mL) in the ascitic fluid and concurrent evidence of fungal growth in cultures. *Candida* species, primarily *Candida albicans*, remain the most frequently identified fungal pathogens in infectious ascitic samples (Tariq et al., 2019). Nevertheless, the differentiation between active infection processes caused by *Candida* species in these samples and mere colonization can be challenging, which complicates the decision of starting antifungal therapy (Carneiro et al., 2011; Yan et al., 2020). The intricate relationship between these findings and patient prognosis intensifies this uncertainty.

The precise identification and classification of fungal pathogens in ascitic fluid is relevant for directing antifungal treatment and refining therapeutic strategies. Conventional culture-based diagnostic methods may, depending on the setting, be both slow and insensitive in ascitic infections: they often take more than two days and report a relative low positivity rate for fungal infections (Arvanitis et al., 2014). Consequently, the diagnosis of ascitic fluid fungal infection leans heavily on clinical assessments, and antifungal treatments are predominantly empirical in nature. This may prolong treatment durations and diminishes success rates (Li et al., 2021).

Culture-independent approaches, such as next-generation sequencing (NGS), have revolutionized our ability to identify a wide array of fungal species, which conventional diagnostics often struggle to cultivate (Irinyi et al., 2016; Kidd et al., 2020). Recent studies have reported the successful use of the ribosomal internal transcribed spacer (ITS1/2) region as a target for the identification of fungi, yet due to the heterogeneity of primers and sequencing workflows used in these reports and the inter- and intraspecies genetic diversity of the fungi associated with various infections, optimized workflows have to be established and evaluated for different pathologies and sample types (Schoch et al., 2012; Irinyi et al., 2015).

This study focuses on detecting and identifying fungal species in ascitic fluid, aiming to gauge NGS’s potential in delivering a faster and more sensitive diagnosis of ascitic fungal infections. By correlating these findings with patients’ clinical outcomes, we endeavour to provide a basis to fine-tune therapeutic interventions in critical care scenarios.

## 2 Methods

### 2.1 Study design and ethics statement

**Figure 1:**
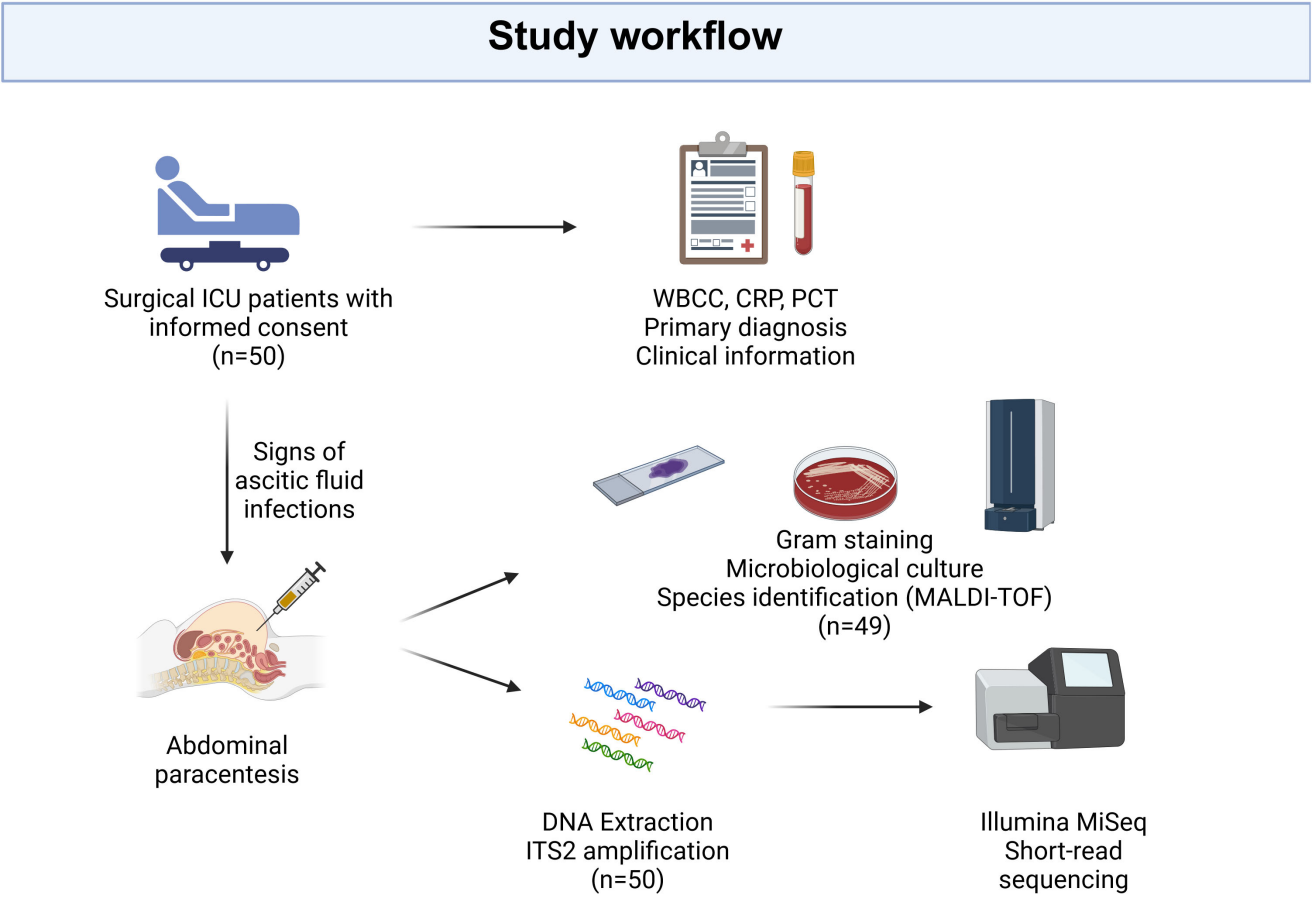
Study design. Patients displaying symptoms of abdominal fluid infection, who underwent paracentesis at the surgical intensive care unit of Freiburg University Medical Center and met the inclusion criteria, were enrolled in the study after obtaining informed consent. Ascites fluid was collected for conventional culture-based microbiological diagnostics, while the remaining fluid was used for DNA extraction and subsequent ITS2 amplification and sequencing. Clinical information, encompassing the primary diagnosis, white blood cell count (WBCC), C-Reactive Protein (CRP), and procalcitonin (PCT), was retrieved from electronic health records. “Created with BioRender.com”.

The study was carried out in the Medical Center of Freiburg University surgical intensive care unit between October 2019 and March 2021. In patients who had undergone abdominal paracentesis due to clinical suspicion of ascitic infection, excess ascitic fluid after conducting standard microbiological diagnostic analysis (at least 5 ml) was immediately frozen at -80°C for metagenomic analysis. An overview of the study design can be seen in **Figure 1**. The study was approved by the Ethics Committee, Medical Centre - University of Freiburg, (registration number 246/19), and registered in ClinicalTrials.gov (NCT04131751). Written informed consent was provided by all participants or their legal representatives in accordance with the Declaration of Helsinki.

### 2.2 Clinical information acquisition

Clinical features were obtained from the electronic health record, with a thorough examination of medical charts to identify antimicrobial prescriptions and assess alcohol and nicotine consumption. Within a ±7-day window surrounding the abdominal paracentesis, we documented levels of various inflammation biomarkers such as white blood cell count (WBCC), C-Reactive Protein (CRP), and procalcitonin (PCT).

### 2.3 Microbiological culture-based methods and microscopy

As part of standard care, ascitic fluid samples were examined microscopically (Gram staining, detection of granulocytes, bacteria, and fungi) and plated on different cultural media such as Columbia blood (Thermo Scientific™ Oxoid™, Wesel, Germany), chocolate blood, MacConkey, yeast extract cysteine blood agar plates (HCB), and (Can2) when diagnosis of fungal infections is requested, followed by incubation for at least 48 hours under aerobic and anaerobic conditions. Inoculated brain heart infusion broth with 0,093% (w/v) agar was incubated for five days. Identification of growing microorganisms was obtained by matrix-assisted laser desorption ionisation-time-of-flight mass spectrometry (MALDI-TOF, Bruker Daltonics, Bremen, Germany).

### 2.4 Microbial genomic DNA preparation

Microbial DNA from human fluid samples is routinely isolated in our lab using a protocol modified from the QIAamp DNA Mini Kit (Qiagen GmbH, Hilden, Germany) that we have previously reported (Goelz et al., 2021). To improve the quality of isolated fungal DNA, a modified protocol with the High Pure PCR Template Preparation Kit (Roche, 11796828001) was established. In brief, the microorganisms were pelleted by centrifugation for 10 min at 5,000 xg. Pellets were resuspended in 400µl Tissue Lysis Buffer (Roche, 11796828001), and microbial cells were disrupted using bead beating lysis tubes containing zirconium silicate grinding beads (Thermo Fisher Scientific, Germany) on the Precellys Evolution homogenisator for a round of 1 min bead beating at 6,000 rpm. Samples were then incubated for 15 min at 95°C, then 40µl of Proteinase K were added and samples were incubated at 56°C overnight in a thermomixer at 1,000 rpm. Samples were then centrifuged at 6,000 xg for 1 min and supernatant was collected in a new 1.5 ml Eppendorf tube. The sample was then further handled following the manufacturer’s protocol.

### 2.5 ITS2 Amplicon library construction and sequencing

An ITS2 amplicon library preparation for Illumina sequencing was constructed as previously described (Wetzel et al., 2024) according to the dual indexing strategy of Kozich et al. (2013) using the gITS7 (forward: GTGARTCATCGARTCTTTG) described in Ihrmark et al. (2012) and ITS4ngs (reverse: TTCCTSCGCTTATTGATATGC) primers (described in Tedersoo et al., 2014), which anneal to the 5·8S and LSU rRNA genes flanking the ITS2 region (Tedersoo et al., 2015). PCR amplification was done under the following conditions: 2 min at 95 °C; 40 cycles of 10 s at 95 °C, 30 s at 56 °C, and 30 s at 72 °C; final extension for 5 min at 72 °C, using the Qiagen UCP Multiplex PCR Kit (Qiagen GmbH, Hilden, Germany). In parallel to DNA-extraction- and PCR-negative controls, a standard bacterial and fungal mock community containing *Saccharomyces cerevisiae* and *Cryptococcus neoformans* (Zymo Research, Irvine, CA), and selected cultured fungal samples (*C.albicans* and *S.cerevisiae*) were used as positive controls in all PCRs and the sequencing run. PCR products were enzymatically purified and barcodes containing Illumina sequencing adapters were added in a second PCR reaction using the Quick-16S NGS Library Prep Kit (Zymo Research, Irvine, CA). PCR products were quantified on a 1.5% agarose gel and Qubit 4.0. fluorometer (Thermo Fisher Scientific, Waltham, MA, USA), then pooled to generate equimolar sub-pools. Pooled libraries were subjected to a DNA purification step using Agencourt AMPure XP beads (Beckman Coulter, Woerden, The Netherlands) then were quantified on Agilent 2100 bioanalyzer (Agilent Technologies, Palo Alto, Calif.). The final library was sequenced using the MiSeq v2 reagent kit (500 cycles) (Illumina Inc., San Diego, CA, USA) on a MiSeq system with 25% PhiX spike-in.

### 2.6 Denoising and inference of amplicon sequence variants

FastQC (Babraham Bioinformatics) and MultiQC (Ewels et al., 2016) were employed to assess the quality of the raw Fastq files (**Supplementary Figure 1**). Further trimming and denoising of short-reads was done using the variation of the DADA2 (Callahan et al., 2016) analysis pipeline for ITS-Sequencing. In the second filtering step using *filterAndTrim*, various values for the *maxEE* and *truncQ* parameters were tested on positive control samples. A value of 1 was selected for both parameters, as it minimized the detection of non-expected species while still successfully detecting the expected ones (**Supplementary Figure 2**). No fixed length trimming was applied, with a minimum length requirement of 20 bp. Amplicon sequence variants (ASVs) were extracted only from forward reads to preserve reads otherwise lost during merging and due to enhanced recovery of the expected species in the positive controls (**Supplementary Figures 2**, **3**).

### 2.7 Taxonomy assignment

The taxonomy assignment was conducted against the UNITE+INSD database for fungi version n° 9.0 (Abarenkov et al., 2023) using a local BLAST (Altschul et al., 1990) search with a maximum of 10 target sequences, a percentage of identical matches >95% and a minimum alignment length of 10 bp. Any ASVs lacking assignment at the phylum level were discarded. For the remaining ASVs that did not reach species-level assignment, a second BLAST search with the same parameters was carried out against the NCBI NT database to achieve a clearer taxonomy level assignment (Sayers et al., 2021). The NCBI NT database was accessed on October 2023 from the NCBI FTP site.

### 2.8 Downstream analysis, data visualization and statistics

Downstream analysis was performed in R version 4.3.1. Contaminants in library preparation were identified using the the “decontam” Bioconductor package by analyzing the taxa frequencies in the true samples and negative controls (Davis et al., 2018). For sample visualization, functions implemented in the R packages phyloseq (Callahan et al., 2016) and ggplot2 (Wickham, 2009) were employed. Statistical analysis and graph generation were conducted using the stats, corrplot and ggstatsplot packages. Data normality was assessed using the Shapiro test. Continuous variables with a non-normal distribution were compared using the Kruskal–Wallis test and the Mann–Whitney U test, while those with a normal distribution were compared using One-Way ANOVA. Categorical variables were compared using Fisher’s exact test, followed by *post hoc* pairwise Fisher exact tests.

### 2.9 Clinical interpretation of microbiological results

All results were interpreted by two experienced clinical microbiologists for clinical relevance and identification of non-pathogenic skin flora or potential contaminants, and potential impact on patients’ therapy was discussed in a panel of clinical microbiologists and treating surgical intensive care attending physicians.

## 3 Results

### 3.1 Clinical features of the study cohort

The final study cohort comprised 50 patients who had suspected ascites infection and were being treated in the University Clinic surgical intensive care unit (ICU). The median age was 64.5 years (range 28-98 years), and the majority of them (33/50) were male. Their underlying diseases were cancer (58%), peritonitis (34%), intestinal ischemia (16%) and cirrhosis (8%). The median values for leukocytes, procalcitonin (PCT) and C-reaction protein (CRP) levels were 15,000/µl, 1.39 ng/L and 125.8 mg/L, respectively. The median ICU-stay was 4 days after paracentesis (range 0-38), and the median hospital stay was 15 days after paracentesis (range 0-86). The in-hospital mortality within six days after sample collection was 10% (5/50). The demographics and comorbidities of the study cohort are shown in **Supplementary Table 1**.

### 3.2 Conventional microbiological fungal detection in ascites patients

Out of the 50 samples, 5 (10%) exhibited fungal growth, and all colonies were identified as *Candida*, *Nakaseomyces* or *Pichia* species (*Nakaseomyces glabratus* was previously known as *Candida glabrata* and *Pichia kudriavzevii* as *Candida krusei*). One sample displayed abundant growth of three Candida species (*N. glabratus, C. dubliniensis, and C. albicans*). Three samples showed varying colony numbers of *C. albicans, C. tropicalis*, and *P. kudriavzevii* respectively, ranging from numerous to sporadic. In the fifth sample, growth of *C. albicans* was observed only after enrichment in the brain heart infusion broth. Culture was not performed in one sample (INT-15).

### 3.3 Generation of ITS2 sequencing data

A total of 50 ascites samples were evaluated for ITS2 sequencing. In previous studies we have shown the sensitivity of the primers of Ihrmark et al. (2012) and Tedersoo et al. (2014) for fungal ITS sequencing, therefore they were used for the amplification of fungal DNA in the ascites samples (Wetzel et al., 2024). In addition to fungal mock community and positive controls, 10 samples showed detectable amplification of the ITS2 gene in gel electrophoresis. The remaining samples lacked evidence of amplification of fungal DNA, including sample INT-46, which had shown growth of *P. kudriavzevii* in the culture. Illumina paired-end sequencing performed on the 50 patients’ samples and controls generated 7,670,917 raw reads, averaging 136,981 reads per sample. **Supplementary Figure 1** shows the overall quality and mean Phred scores (Q-score) of the samples. All fungal species from the positive controls were successfully recovered (**Supplementary Figure 3**).

### 3.4 Evaluation of ITS2 sequencing output compared with standard microbiology culture results

**Figure 2:**
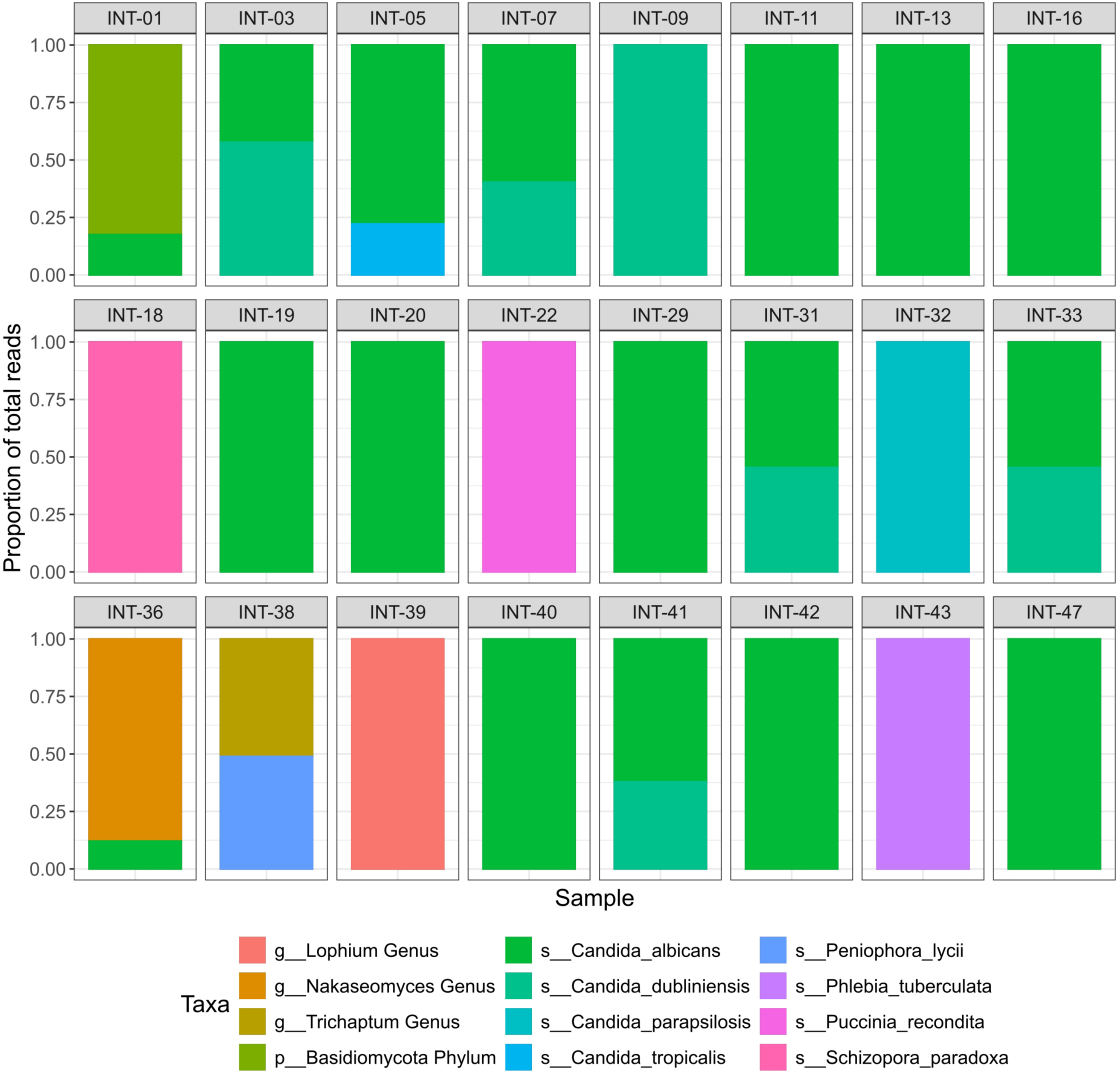
Fungal taxa identified with ITS2-sequencing. Relative abundance of fungal taxa identified in ITS2 sequenced samples after filtration. Samples that retrieved no fungal reads after filtering are not shown. The Y-axis represents the proportion of total reads and colors represent the identified taxa.

After excluding the ASVs identified as library contaminants and those that could not be assigned to any fungal phylum, based on the taxonomy assignment strategy detailed in the methods, 231 ASVs remained for further analysis. With respect to these filtered ASVs the sample with the highest read count contained 294,856 reads, with a median read count of 10,381. The ASV with the most reads in a single sample had 245,252 reads, while the average was 5,524. After merging ASVs belonging to the same taxa, the highest taxa count in a sample was 245,336, with an average of 9,249. The distribution of read counts can be seen in **Supplementary Figure 4**. Given the diagnostic nature of our study, we aimed to minimize irrelevant findings and focus on taxa most likely to be clinically consequential. Therefore, we implemented a cut-off of 10,000 reads per species to ensure that only sufficiently abundant species were considered potentially relevant. After this filtering, 12 taxa remained, of which one was only assigned to the Phylum level (Basidiomycota), three were only assigned to the Genus level and eight were assigned to the species level (**Figure 2**; **Supplementary Figure 5**). The identified fungi were subsequently categorized into facultative pathogenic (e.g. all *Candida* species) or likely contaminant (e.g. *Peniophora lycii*). Of the 11 fungal taxa identified to the genus level, six were classified as probably contaminant, while five taxa were classified as facultative pathogenic (**Table 1**; **Supplementary Table 2**).

**Table 1:** Facultative pathogenic fungi detected with ITS2 sequencing compared with routine microbiology culture results.

Sample_ID	ITS2 PCR	ITS2 Sequencing results	Reads	Culture	Cultured species*
**INT-01**	+	*Candida albicans*	12939	-	-
		*Basidiomycota*	60764		
**INT-03**	+	*Candida dubliniensis*	34243	–	–
		*Candida albicans*	24342		
**INT-05**	+	*Candida albicans*	124944	+	*Candida tropicalis +*
		*Candida tropicalis*	35661		
**INT-07**	**+**	*Candida albicans*	22819	–	–
		*Candida dubliniensis*	15439		
**INT-09**	-	*Candida dubliniensis*	57210	-	-
**INT-11**	*+*	*Candida albicans*	59734	*-*	*-*
**INT-13**	+	*Candida albicans*	245336	-	**-**
**INT-16**	*-*	*Candida albicans*	12839	*-*	*-*
**INT-19**	-	*Candida albicans*	21559	-	**-**
**INT-20**	*-*	*Candida albicans*	60022	*-*	*-*
**INT-29**	-	*Candida albicans*	14444	-	**-**
**INT-31**	–	*Candida albicans*	29871	+	*Candida albicans* after enrichment
		*Candida dubliniensis*	24911		
**INT-32**	-	*Candida parapsilosis*	11063	-	**-**
**INT-33**	–	*Candida albicans*	40186	–	–
		*Candida dubliniensis*	33501		
**INT-36**	+	*Nakaseomyces*	220234	+	*Nakaseomyces glabratus* +++ (also in blood culture)
		*Candida albicans*	30390		*Candida albicans* ++
					*Candida dubliniensis* ++
**INT-40**	*+*	*Candida albicans*	135539	*-*	*-*
**INT-41**	+	*Candida albicans*	182489	+	*Candida albicans* ++
		*Candida dubliniensis*	111650		
**INT-42**	*-*	*Candida albicans*	10293	*-*	*-*
**INT-46**	-	*-*	-	+	*Pichia kudriavzevii +*
**INT-47**	*+*	*Candida albicans*	78675	*-*	*-*

*The number of “+” symbols indicate the semi-qauntative scoring of the fungal growth in culture in which: + = Scattered growth; ++ = Numerous growth; +++ = Abundant growth.

From 50 samples 24 showed evidence of fungal taxa, from which 19 samples had ASVs identified as facultative pathogenic (**Table 1**). In 29 samples there was no detection of facultative pathogenic species in ITS2 sequencing, consistent with the negative culture results. All isolates that were identified by culture were confirmed through sequencing, except for two isolates from two different samples. One was a *P. kudriavzevii* isolate from sample INT-46, which showed no ITS2 amplification. The other was a *C. dubliniensis* isolate from sample INT-36. Although this sample had 204 reads aligning with *C. dubliniensis*, they were excluded in the final filtering step.

### 3.5 NGS-based fungal detection correlates with patients’ outcome

**Figure 3:**
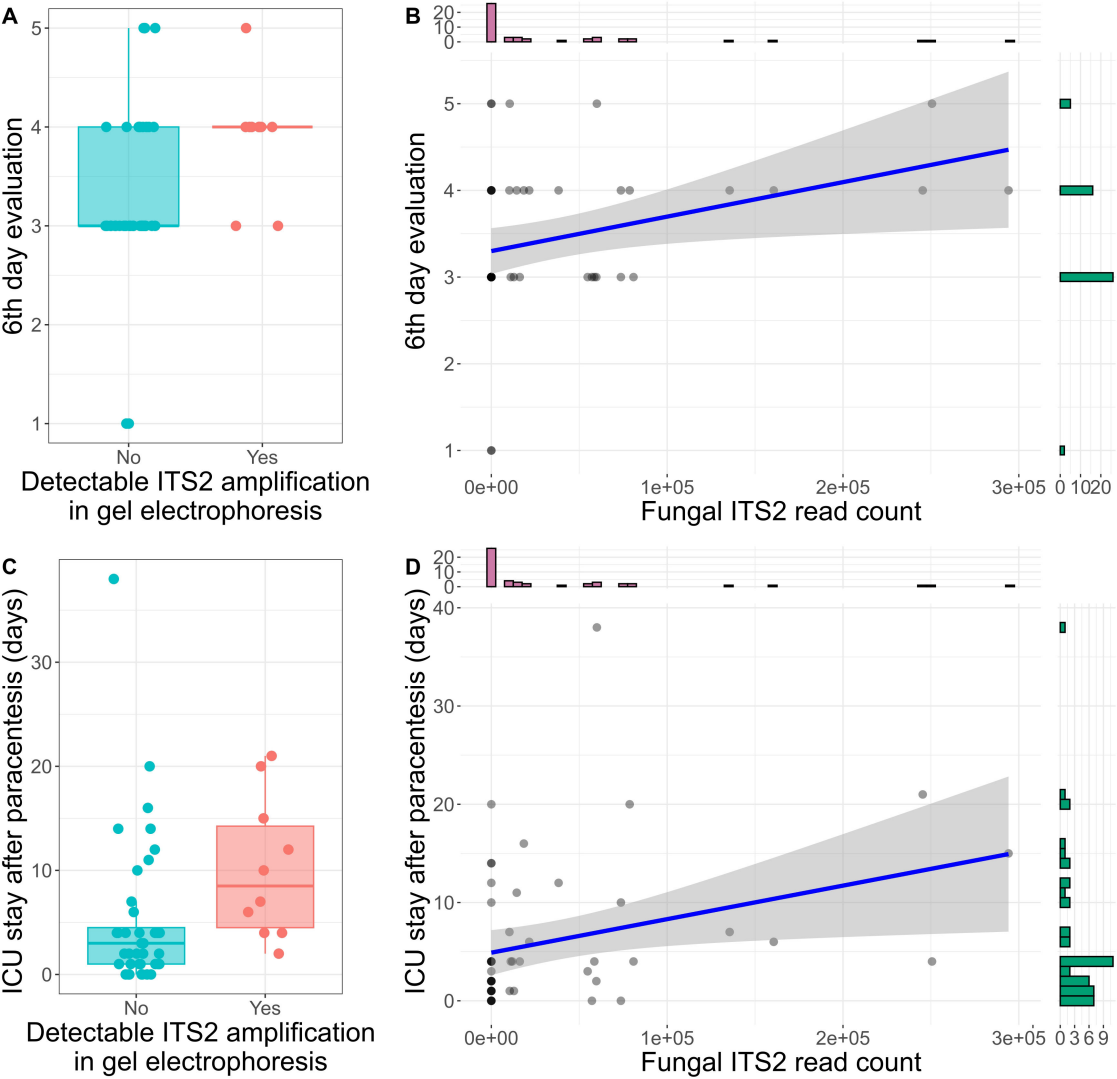
ITS2 sequencing is associated with ascitic patients’ outcome. **(A)** Box plot displaying the score of the sixth-day evaluation in patients with and without detectable ITS2 amplification in gel electrophoresis. In Boxplot 3A, 3C each dot represents one sample. The central line signifies the median score. Boxes cover the interquartile range (IQR), from the 25th to the 75th percentile, representing the middle (50%) of the data. The whiskers stretch to the minimum and maximum values within 1.5 times the IQR from the quartiles. **(B)** Spearman correlation of fungal read counts and the sixth-day evaluation score. The histograms on the x and y-axis represent the number of samples in each group of read counts or sixth-day evaluation score respectively. **(C)** Box plot displaying the length of ICU stay in patients with and without detectable ITS2 amplification in gel electrophoresis. **(D)** Spearman correlation of fungal read counts and the length of ICU stay. The histograms on the x and y-axis represent the number of samples in each group of read counts or ICU stay length respectively.

Six days following paracentesis, patient status and recovery was evaluated using a 1-5 scale. A score of 1 represented hospital release, 2 indicated transfer to a non-tertiary care facility, 3 indicated transfer from intensive care to a regular hospital ward, 4 indicated an ongoing need for intensive care, and 5 indicated patient death. Patients with samples containing higher fungal DNA load -as represented by the detection of the ITS2 amplification products in gel electrophoresis- had a significantly higher score on the sixth-day evaluation (median 4, range 3-5, n = 10) compared to those without detectable ITS2 amplification (median 3, range 1-5, n = 39) (Mann-Whitney U test: z = 2.28, p = 0.02, r = 0.43, n = 49, **Figure 3A**). Additionally, there was a significant positive correlation between the number of ITS2 reads and the sixth-day evaluation score (Spearman’s rank correlation: ρ = 0.36, p = 0.01, n = 49, **Figure 3B**).

Detectable ITS2 amplification in gel electrophoresis was also associated with a longer ICU stay (median 8.5 days for detectable ITS2 amplification vs. 3 days for no amplification) (Mann-Whitney U test: z = 2.7, p = 0.007, r = 0.55, n = 50, **Figure 3C**). Furthermore, the ITS2 read count correlated positively with the duration of ICU stay (Spearman’s rank correlation: ρ = 0.39, p = 0.005, n = 50, **Figure 3D**). Inflammatory markers measured closest to the time of sample collection, including leukocytes, C-reactive protein (CRP), and procalcitonin, did not show significant associations with fungal culture or ITS2 sequencing reads.

**Figure 4:**
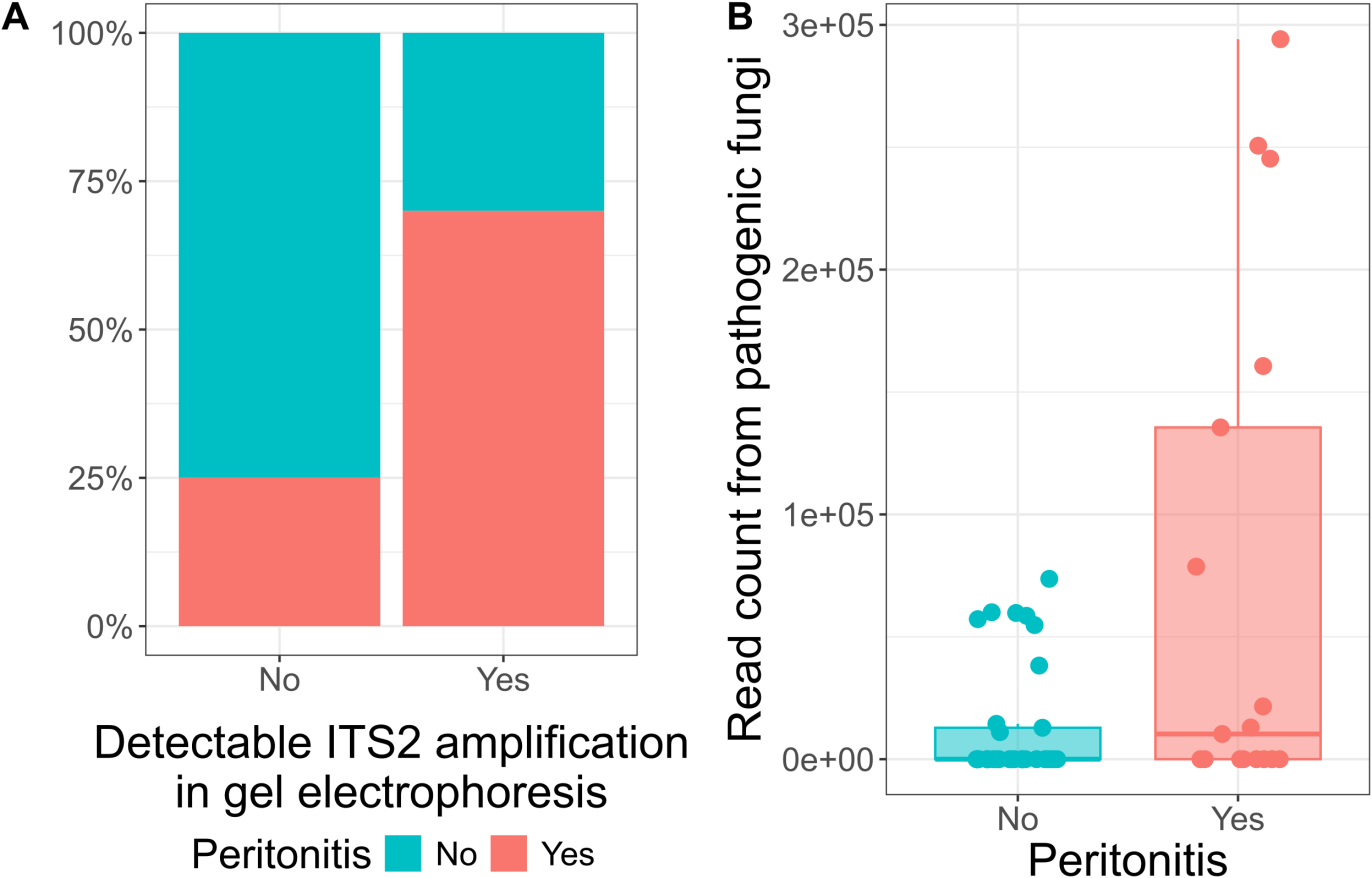
Detection levels of ITS gene in ascites patients is associated with peritonitis. **(A)** Bar plot displaying the percentage of presence of peritonitis cases in patients with and without detectable ITS2 amplification in gel electrophoresis. From the patients with detectable ITS2 amplification, 70% (7/10) developed peritonitis. **(B)** Box plot displaying the read count from pathogenic fungi in patients with and without peritonitis. Each dot represents one sample. The central line signifies the median score. The box covers the interquartile range (IQR), from the 25th to the 75th percentile, representing the middle (50%) of the data. The whiskers stretch to the minimum and maximum values within 1.5 times the IQR from the quartiles.

Next, we investigated whether patients’ clinical manifestations associated with ITS2 sequencing results. Patients with detectable ITS2 amplification on gel electrophoresis developed peritonitis more frequently (Fisher’s exact test, p = 0.02, odds ratio = 6.7, **Figure 4A**). In addition to that, pathogenic fungal reads were higher in patients with peritonitis (Mann-Whitney U test: z = 1.94, p = 0.05, r = 0.30, n = 50, **Figure 4B**). No significant associations were found between fungal ITS2 reads and conditions such as cancer, intestinal ischemia, or cirrhosis. However, 60% (6/10) of patients with detectable ITS2 amplification had either cancer or intestinal ischemia as underlying conditions.

### 3.6 Antifungal therapy of ascites patients

Antifungal therapy was associated with fungal growth (Fisher exact test: p-value 0.03, odds ratio 9.6, CI 0.9-143.09), but not with detection of pathogenic fungi in ITS2 sequencing (z = 1.59, p = 0.11, r = 0.32, n = 45, **Supplementary Figure 6**). Out of the 19 patients with identifiable pathogenic fungal ITS2 reads in sequencing, antifungal therapy was administered to five individuals. Two of them received Anidulafungin and Fluconazole following the growth of *C. tropicalis* (INT-05) and *C. albicans* (INT-41) in culture, respectively. The patient (INT-36), who exhibited growth of *N. glabratus, C. albicans* and *dubliniensis* in ascites culture, had positive blood cultures for *N. glabratus*. Micafungin was promptly administered after sample collection and four days later liposomal Amphotericin B was added. Two patients (INT-01 and INT-42) were treated with Fluconazole and Caspofungin, respectively, following the detection of *C. albicans* in culture of a previous ascites samples. Thirteen patients did not receive any antifungal treatment, and information about treatment was not available for one individual.

Among the 31 patients without pathogenic fungal ITS2 reads, information about antifungal therapy was available for 27. Among these, three received antifungal therapy. One case was attributed to the identification of *Candida* species in previous samples, and in two cases the treatment was initiated empirically.

## 4 Discussion

Ascitic fluid fungal infection represents a severe complication associated with adverse clinical outcomes and a marked surge in mortality, particularly among patients in critical care units. Accurate microbiological diagnostics and the identification of the causative fungi are important in improving patient outcomes. Our study shows the benefits of employing next-generation sequencing in diagnosing fungal ascitic infections among ICU patients. Various fungal species, typically under-recognized, were detected in the ascitic fluid, suggesting potential pathogenic significance.

Of the 50 samples included in our study, 10% (5 samples) were positive for *Candida* species, *N. glabratus*, or *P. kudriavzevii* in culture. Sequencing confirmed the identification of all isolates found in culture, except for two isolates from two samples. In the case of sample INT-46, laboratory contamination cannot be ruled out, particularly since no ITS2 amplification was observed in gel electrophoresis. Patient INT-36 showed evidence of *N. glabratus* and *C. albicans* in both culture and sequencing, along with candidemia due to *N. glabratus*. However, *C. dubliniensis*, which had grown in culture, was not detected in ITS2 sequencing, as the low number of reads was filtered out. This low read count could be attributed to the overwhelming presence of *N. glabratus* in both the culture and sequencing. Additionally, the absence of *C. dubliniensis* but presence of *C. albicans* in our positive controls raises the possibility that reads from *C. dubliniensis* may have been misaligned to *C. albicans*. This possibility should be considered in future studies. However, the susceptibility profiles of the two species are quite similar, and may have minimal effect on the patient’s treatment (Ayadi et al., 2020). Overall, this suggests that culture results are reasonably consistent with sequencing findings. On the other hand, ITS2 sequencing identified facultative pathogenic fungi in the ascitic fluid of 19 patients, 15 of whom would not have had fungi detected through culture alone. The identified facultative pathogenic fungi included *Nakaseomyces* and *Candida* species, such as *C. albicans*, which is the most commonly reported fungal species in ascitic fluid cultures, both in the literature (Tariq et al., 2019), and in our study group.

The majority of the patients with positive ITS2 sequencing reads had cancer or intestinal ischemia, conditions that lead to increased intestinal permeability (Vollmar and Menger, 2011; Bindels et al., 2018), providing the opportunity for the fungi to translocate to the peritoneal cavity (Bremmer et al., 2015). Additionally, detectable ITS2 amplification was associated with the development of peritonitis. We have previously reported the findings regarding the bacterial infections corresponding to this cohort (Goelz et al., 2021). The concomitant detection of bacteria in some samples suggests the possibility that bacterial infections also contribute to the development of peritonitis, leading to an elevated level of intestinal permeability and subsequent fungal translocation into the peritoneal cavity. This dual presence of bacteria and fungi in some cases makes it challenging to definitively determine whether fungal translocation is the primary cause of peritonitis or rather a consequence of the bacterial infection, nevertheless the significant correlation between peritonitis and ITS2 results suggest a potential role for fungi in the development of the complication.

Our study reveals a clear association between positive ITS2 sequencing and an unfavorable prognosis, as indicated by assessments conducted on the sixth day and the length of ICU stay. Previous studies extensively highlighted the high mortality rates associated with spontaneous fungal peritonitis (Bremmer et al., 2015; Tariq et al., 2019; Jiang et al., 2023). This elevated risk of adverse clinical outcomes may be the result of low awareness levels among clinicians, especially compared to spontaneous bacterial peritonitis, and the extended duration needed for fungal growth and treatment (Bremmer et al., 2015). Our findings suggest that ITS2 sequencing might aid in quicker diagnosis -possibly within 48 hours if well established- of these patients compared to traditional culture methods, especially in cases where no fungal growth is observed and thus reduce the elevated mortality. Among patients with positive ITS2 sequencing, only five patients positive in culture, either in the same or in previous samples, received antifungal therapy. ITS2 sequencing might help select the individuals who should receive antifungal therapy and guide the targeted management of fungal infections following species identification.

One of the main goals of the study was to improve and to standardize sequencing workflows for the analysis of fungal infections of human ascitic fluid. Isolation of fungal DNA was improved by optimizing a protocol that favors the isolation of fungal genomic materials following disruption of their thick cell wall. For the taxonomy assignment, a BLAST-based approach was followed, as it achieved better species-resolution in a community of medically relevant fungi in comparison with the RDP naive Bayesian classifier implemented in DADA2 (Rolling et al., 2022). Reaching species-level annotation is relevant for targeted treatment given the varying antifungal resistance patterns among species within a genus (Lee et al., 2021).

Even though species level identification based on Illumina short read sequencing can be quite complex, we could correctly identify the species of the positive controls that were included in the study. However, not all relevant fungal species for human infections were included in the positive controls, and thus the sensitivity of this workflow should be further validated for species level classification in future studies. In our samples, species level identification could be achieved to a good extent, even though not in all taxa. The only relevant taxa that could not be identified to the species level was the one corresponding to the genus *Nakaseomyces*. The important species for human infections from this genus are the ones belonging to the *C. glabrata* clade (*N. glabratus, N. brancariensis and N. nivariensis*), which all have reported high minimum inhibitory concentrations (MICs) for fluconazole, also extending to other azoles, and lower MICs for echinocandins. Therefore, the identification of a fungal isolate as a member of the *C. glabrata* clade would be mostly sufficient to guide empirical antifungal therapy with an echinocandin, until potential susceptibility testing from a grown culture can be obtained (Fujita et al., 2007; Bishop et al., 2008; Borman et al., 2008).

Although exploratory in nature, this study highlights the significance of ascitic fungal infections, particularly in ICU patients, and underscores the potential of NGS methods to enhance their diagnosis. However, further evaluation of these NGS methods for fungal detection in larger patient cohorts, along with optimization of antifungal therapy based on the findings, is crucial before their full integration into standard diagnostic workflows.

## 5 Conclusion

Our study emphasizes the relevance of introducing NGS-based techniques as a diagnostic tool for fungal infections, especially for patients in intensive care units, who have a higher prevalence of concurrent conditions and an elevated risk of death. We provide a workflow for fungal detection based on ITS2 amplification and sequencing. However, the integration of these techniques into everyday microbiology diagnostics requires thorough standardization and the careful interpretation of sequencing results for each patient according to the clinical presentation.

## Data Availability

The datasets presented in this study can be found in online repositories. The names of the repository/repositories and accession number(s) can be found below: https://www.ebi.ac.uk/ena, PRJEB72809, run accession numbers ERR13702674-ERR13702729.
