# Supplementary material for: Enhancing ascitic fungal infection diagnosis through next-generation sequencing: a pilot study in surgical ICU patients
Source: Front Cell Infect Microbiol. 2024 Nov 1;14:1441805. doi: 10.3389/fcimb.2024.1441805 (PMC11564152; doi:10.3389/fcimb.2024.1441805)
Supplement: Supplementary file 1 [file Supplementaryfile1.docx]

Supplementary Material

# Supplementary Figures

**
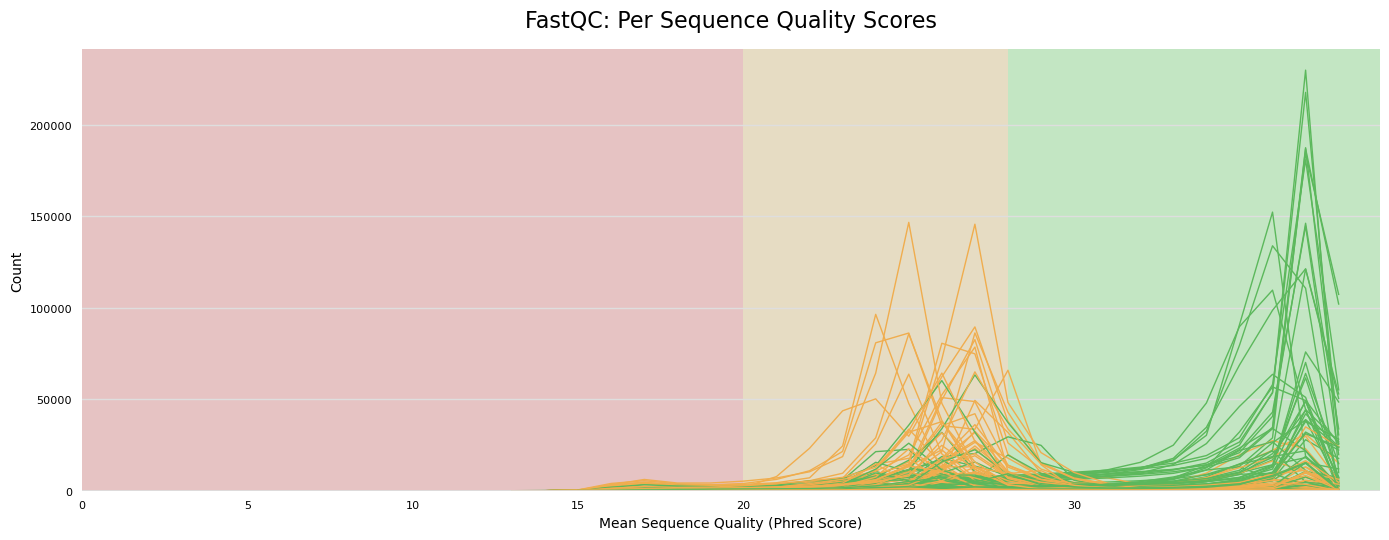
**

**Supplementary figure 1. Quality control of ITS2 raw sequencing reads.** Each line represents the number of reads (Y-axis) with a determined average quality score (X-axis) in the forward or reverse read subset of one sample. The overall quality of the sequencing data has a mean Phred score (Q-score) of greater than 20, which speaks for the good quality of the sequencing data.

**
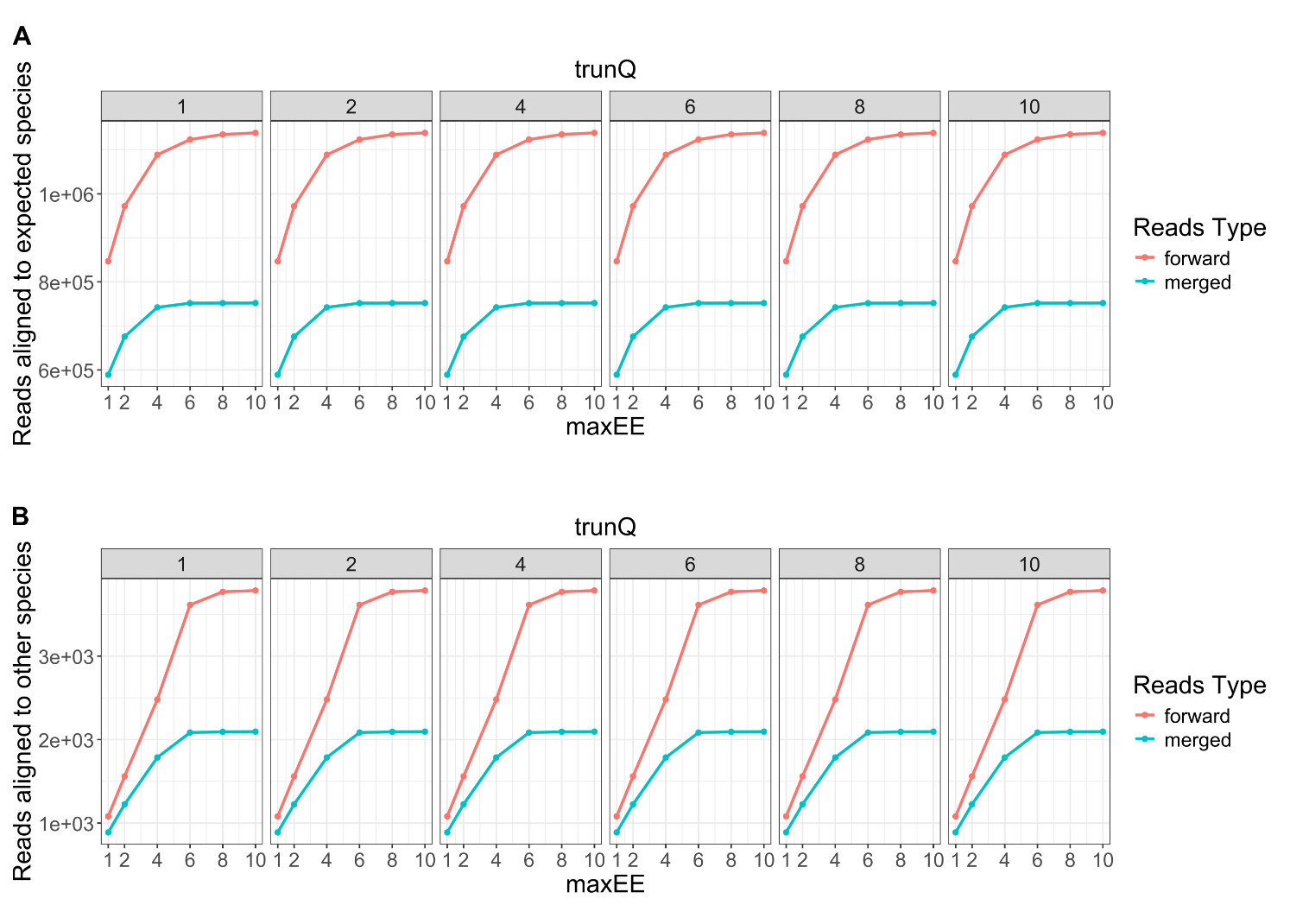
**

**Supplementary figure 2. Evaluation of truncQ and maxEE parameters.** The figure shows the number of reads that aligned to the expected species (**A**) or to non-expected species (**B**) in positive controls. The x-axis represents the maxEE values, while the facets indicate different truncQ values. Blue lines represent reads derived after merging, and red lines represent only forward reads.


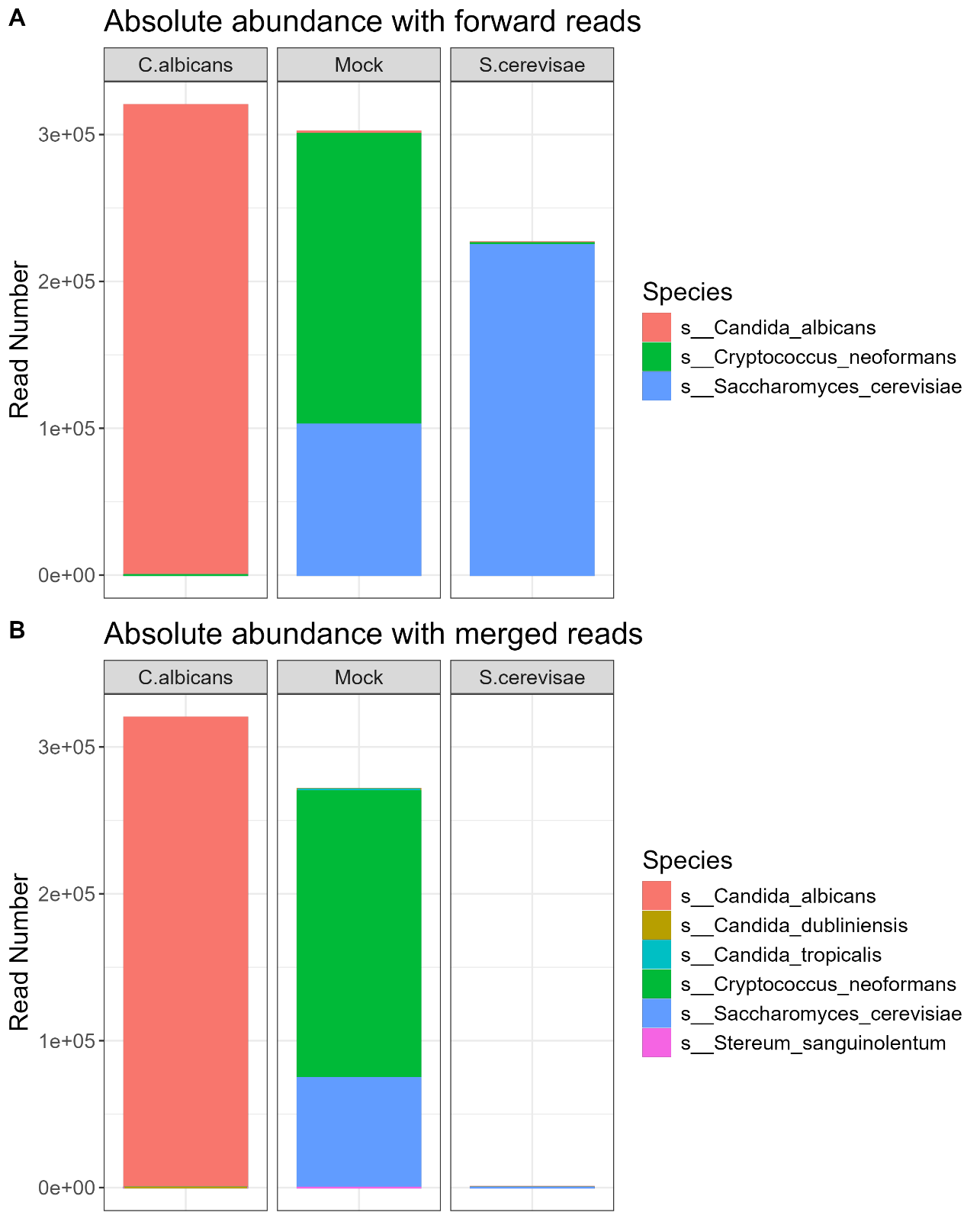


**Supplementary Figure 3. Analysis of positive controls using Illumina ITS2 DNA sequencing.** Absolute abundance of species recovered from *C. albicans* and *S. cerevisiae* isolates, as well as from the mock community containing *S. cerevisiae* and *C. neoformans*, using only forward reads (**A**) or merged forward and reverse reads (**B**). Species representing less than 0.1% of the sample were excluded for clarity. The Y-axis represents the total read count in each sample.

**
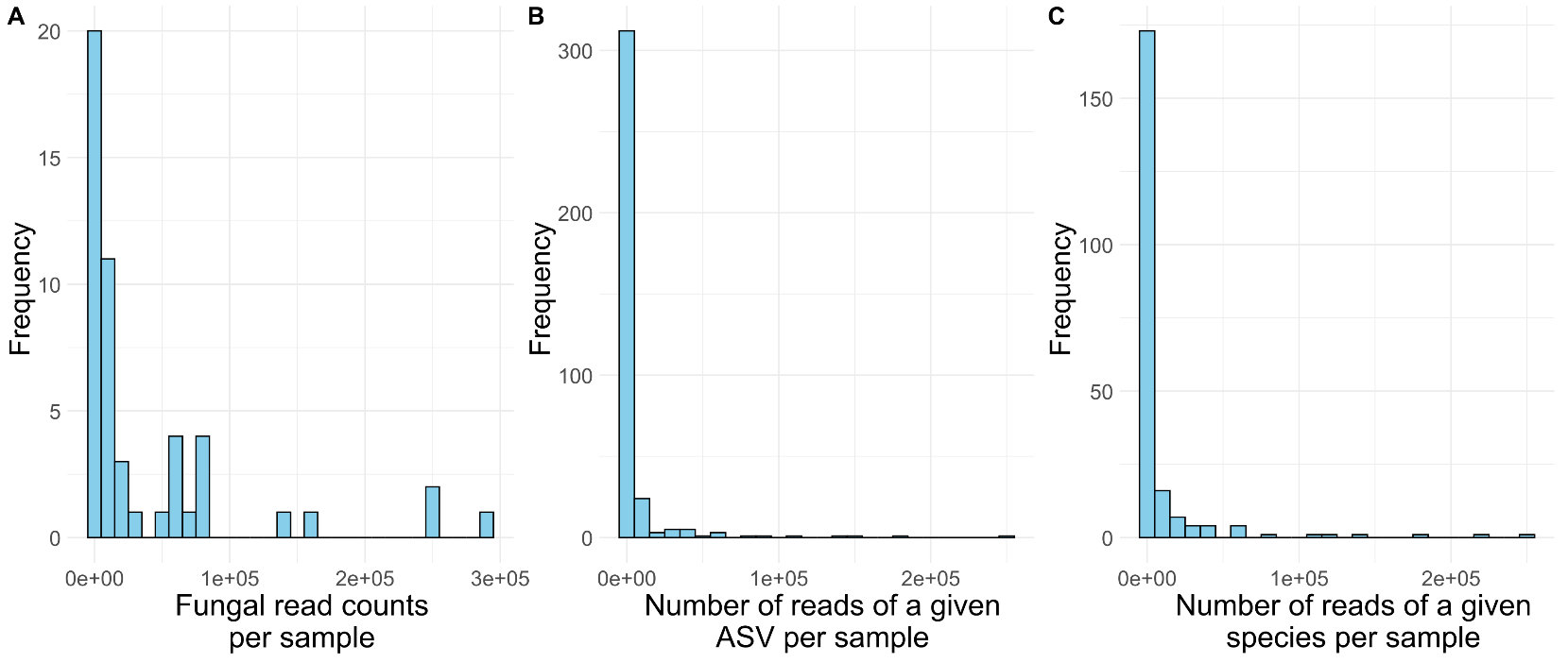
**

**Supplementary Figure 4. Read counts distribution.** The histogram illustrates the distribution of read counts in three contexts: (**A**) total read counts per sample, (**B**) read counts per ASV per sample, and (**C**) read counts per species or lower taxonomic level per sample. Bins are set at intervals of 10,000 reads.

**
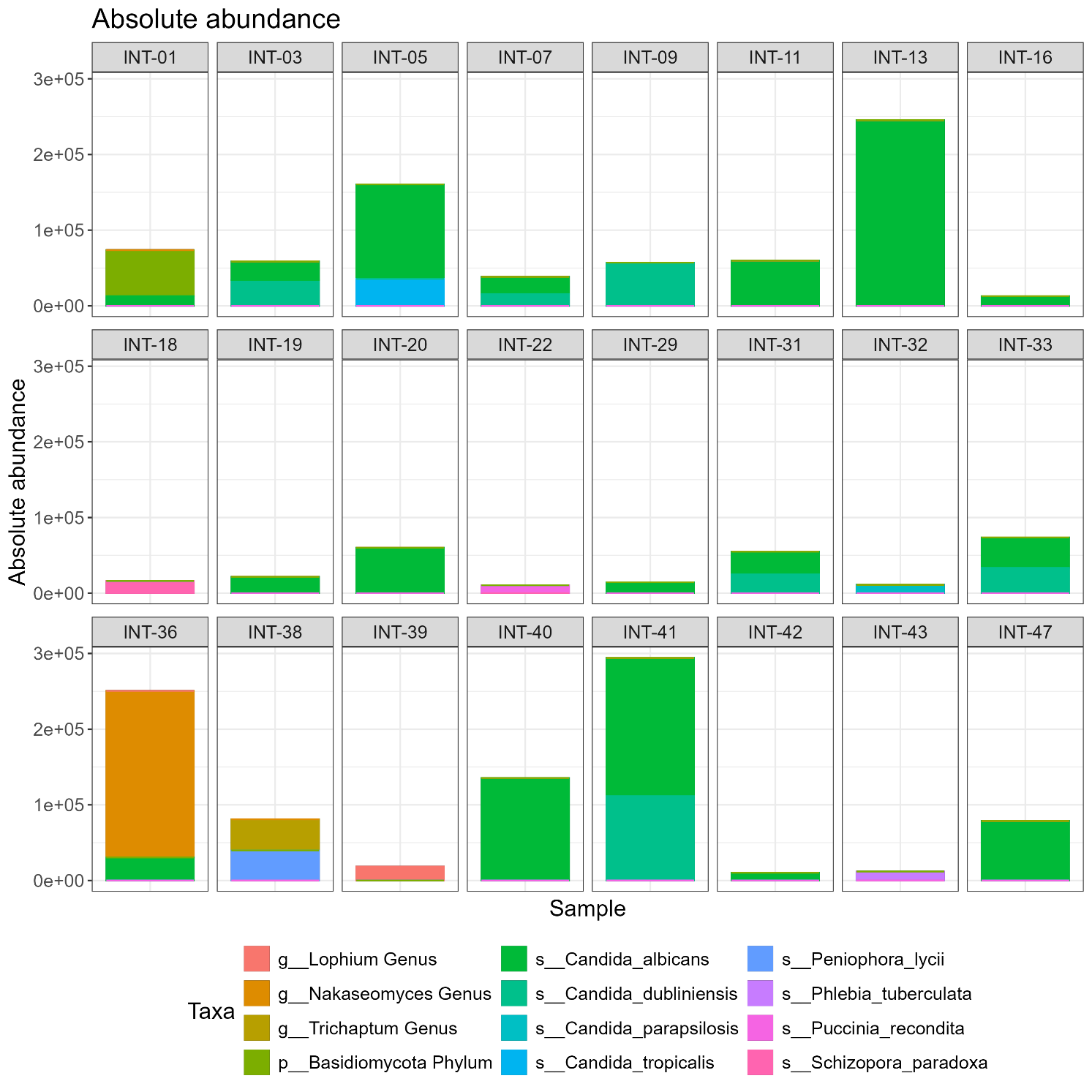
**

**Supplementary Figure 5. Fungal taxa identified in ITS2-sequencing.** Absolute abundance of fungal taxa identified in samples where ITS2-sequencing was performed. Samples that retrieved no fungal reads after filtering are not shown. The Y-axis represents total read number and colors represent the identified taxa.

**
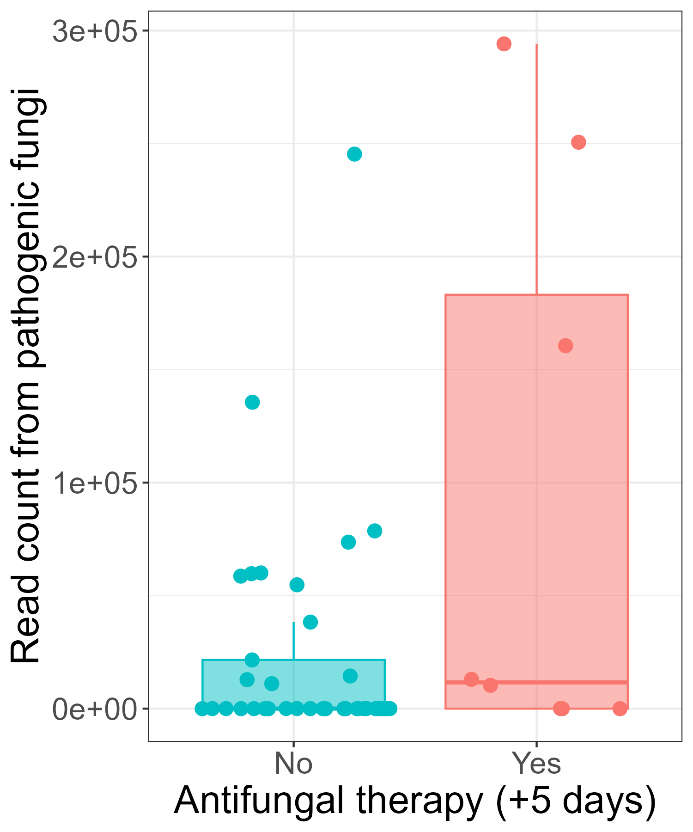
**

**Supplementary Figure 6. Antifungal therapy in patients with read counts aligning to pathogenic fungi.** Box plot illustrating the relationship between ASV read counts from pathogenic fungi and antifungal therapy administered within five days of sample collection. A Mann-Whitney U test revealed no significant differences (z = 1.59, p = 0.11, r = 0.32, n = 45).
